# Posterior short segment pedicle screw fixation for the treatment of thoracolumbar fracture

**DOI:** 10.11604/pamj.2020.35.102.21540

**Published:** 2020-04-07

**Authors:** Mourad Aoui, Nizar Sahnoun, Mohamed Abid, Mahdi Maatoug, Majdi Hsairi, Yosr Hentati, Hassib Keskes

**Affiliations:** 1 Orthopedics and Trauma Department, Habib Bourguiba University Hospital, Sfax, Tunisia; 2 Anesthetic Department, Habib Bourguiba University Hospital, Sfax, Tunisia; 3 Radiology Department, Hedi Chaker University Hospital, Sfax, Tunisia

**Keywords:** Thoracolumbar, fracture, fixation, instrumentation, short segment, osteosynthesis

## Abstract

The choice of the type of stabilization device in the osteosynthesis of dorso-lumbar spine fractures remains a subject of controversy. The present study aims to evaluate the efficiency of short segment in patients suffering post-traumatic thoracolumbar fractures. This study was conducted in the Department of Orthopedic Surgery and Traumatology of the Habib Bourguiba University Hospital, Sfax, Tunisia. All our patients had a spinal osteosynthesis via the posterior approach with a short segment pedicle screw fixation. We established a record of the pre and post-operative data, the functional results in the post-operative stage during the follow-up period and in retrospect according to the Denis Pain Scale, as well as the Oswestry score. The correction was evaluated by determining the relative gain and loss at the last period of retrospect: vertebral kyphosis, regional kyphosis, Gardner Segment Kyphotic Deformity (GSKD), and computed tomography (CT) scan in retrospect to check the quality of the arthrodesis. The average Oswestry score was 14%. Twenty-nine patients had an Oswestry score ≤40%. The relative gain obtained postoperatively was 57.3% for vertebral kyphosis, 67.2% for regional kyphosis and 71.3% for Gardner kyphosis deformity; while the loss of correction at the last follow-up was 0.6° for vertebral kyphosis, 1.5° for regional kyphosis and 0.9° for GSKD. No cases of non-union were reported. The short segment fixation makes it possible to limit operating time, the abundance of bleeding and the aggression of the soft tissues.

## Introduction

Over the last two decades, numerous technical advances in instrumentation and in the knowledge of spinal biomechanics have modified the surgical strategies for the synthesis of spinal-lumbar spine fractures [1]. The choice of the stabilization device type in the osteosynthesis of dorsolumbar spine fractures remains a subject of controversy. Some authors take into consideration only the type of fracture and the degree of its comminution [2], while some others choose the segmental instrumentations according to the patient´s habit [3]. Through our work, we evaluate the short segment pedicle screws fixation of thoracolumbar fractures.

## Methods

We conducted a retrospective, descriptive study over an 8-year period involving 30 patients operated on for a thoracolumbar spine fracture at the Orthopedic Surgery and Trauma Department of the Habib Bourguiba University Hospital in Sfax, between July 2011 and July 2019. All patients were operated on by the same surgeon and with the same technique. All of our patients underwent a spinal osteosynthesis via the posterior short segment instrumentation, and a posterior and postero-lateral graft. In our study, we included patients with: age ≥15, complete preoperative, postoperative and retrospective radiological assessment with a minimum retrospect of 1 year. We established a record determining the clinical and radiological data of each patient. On the clinical level, we specified the general information about the patient and the circumstances of the accident. The record also included the characteristics of the fracture and its consequences on the spinal statics, the neurological status, the pre and post-operative data (operating time, transfusion, neurological release, length of hospital stay, post-operative recovery, and rehabilitation), and the postoperative. Retrospective clinical and anatomical results according to the Denis Pain Scale, the Oswestry score (ODI: Oswestry Disability Index), the Schöber Index and return to work were considered in the report. On the radiological level, we estimated the correction by determining the relative gain and losses at the last follow-up vertebral kyphosis (VK), regional kyphosis (RK) and Gardner Segment Kyphotic Deformity (GSKD). We also performed the study of sagittal balance through the sagittal heel in T9 and the sagittal vertical axis (SVA) measured on an x-ray of the entire spine standing front and in the profile made at the last retrospect. Scannographic analysis was performed to check the quality of the arthrodesis.

## Results

**Figure 1:**
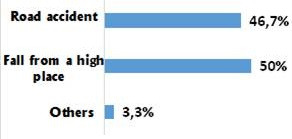
Injury mechanisms

**Figure 2:**
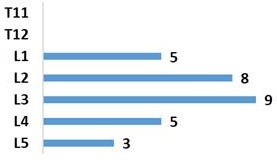
Level of injury

**Figure 3:**
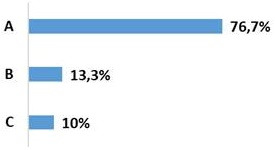
Distribution according to the Magerl classification

Our population included 29 men and only 1 woman. Average age was 32.6 years with a standard deviation of 10.7. The trauma was due to falls from a high place in 50% of the cases (palm tree climbers) and to road accidents in 46.7% of the cases (Figure 1). Associated lesions were found in 56.7% of the cases. Eight cases of poly-trauma and 09 cases of peripheral lesions were noted. Twenty-four patients (80%) had no neurological damage at the time of admission and 6 (20%) had partial neurological damage. A rate of 56% of the fractures were at vertebra L3, L4 and L5 and 44% concerned the thoracolumbar junction (T11, T12, L1 and L2) (Figure 2). The fracture was Type A of Magerl classification in 76.7% of the cases, type B in 13.3%, and type C in 10% of the fractures (Figure 3). The evaluation of the average deformation was about 16.2° for the CV, 10.7° for the CR and 18.2° for the GSKD.

**Figure 4:**
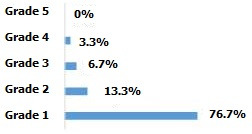
Functional results according to DPS

The mean operating time was 99.3 minutes with a standard deviation of 16.7 minutes. Pre- or postoperative transfusion was necessary in only one patient in our population. A laminectomy was performed in 8 cases (26.7%). The length of the hospital stay was 12.2 days on average and the delay in getting up postoperatively was of 2.9 days in average in non-neurological patients, with extremes ranging from 2 to 5 days. Four patients (13.3%) did not adhere to our rehabilitation protocol. The average decline in our series is 51.1 months with a standard deviation at 24 months. According to the Denis Pain Scale (DPS), 27 patients (90%) had a grade <3 and 3 patients (10%) had a grade ≥3 (Figure 3). The average ODI was 14%. Twenty-nine patients (96.7%) had an ODI score ≤40% and only 1 patient (3.3%) had a score >40% (Figure 4).

**Figure 5:**
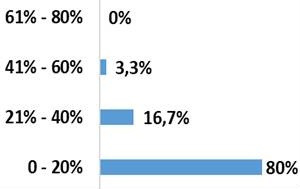
Functional results according to ODI

**Figure 6:**
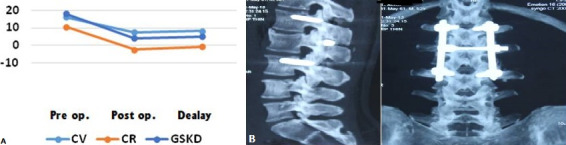
A) evolution of the radiological parameters; B) CT images in favor of the outline of anterior arthrodesis

Twenty-six patients (86.7%) returned to work within 7.9 months and 4 patients (13.3%) stopped working permanently. The average Schöber index was 12.9/10 cm with a standard deviation of 0.6 cm. All patients initially presenting a partial neurological deficit improved by at least one grade according to the Frankel classification, 50% of which showed total neurological recovery. We also deplored a case of sepsis on early material. However, no case of non-union or implant failure was noted. Regarding the radiological results, the relative gain obtained postoperatively was 57.3% for VK, 67.2% for RK and 71.3% for GSKD (Table 1) and the loss of correction at the last follow-up was 0.6° for VK , 1.5° for RK and 0.9° for GSKD (Figure 5). As for the study of sagittal balance, 8 patients (33.3%) were well balanced and 16 patients (66.7%) were unbalanced. In our series, all the fractures were consolidated and no case of implant failure was reported (Figure 6).

**Table 1:** Radiological results

		Mean	Standard deviation
**Relative gain**	**CV**	57,3%	43,1%
	**CR**	67,2%	138%
	**GSKD**	71,3%	48,7%
**Loss of correction**	**CV**	0,6°	1,1°
	**CR**	1,5°	3°
	**GSKD**	0,9°	1,7°

## Discussion

In congruity with the literature [4], the studied series of patients in the present investigation scored a clear male predominance, with 29 men and only 1 woman, the average age of our patients was 32.6 years. Accidents on the public highway were present in two cases. The frequent association of the thoracolumbar spine fracture with other lesions indicates the importance of the causal energy. This association was found in 17 of our patients (56.7%) and it is similar to other series in the literature [3]. According to the classification of Frankel HL *et al.* [5], the absence of neurological deficit classified as Frankel E, was noted in 80% of our patients. Eno J-JT *et al.* [6] found that 68% of patients presented neurological signs at admission. Li X *et al.* [7] found 36% of the patients with neurological signs. Concerning thoraco-lumbar fractures, many classifications have been used in the literature. Some are based on the mechanism and type of fracture, such as the classification of Magerl [8] and that of Denis [9]. Others are based on scores calculated from the neurological state, from the anatomopathological type of the fracture and from the state of the complex posterior ligament, such as the classification of TLICS advanced by Vaccaro A *et al.* [10]. The Load Sharing Scoring is made of a score calculated from the degree of compaction of the vertebral body [2].

According to the Magerl´s classification [8], type A was found in 76.7% of cases. This predominance is found in another series discussing the fractures treated by a short segment fixation for the Dobran series in 92% of the cases [11] and for the Farrokhi series in 87% of the cases [12]. The Load Sharing Scoring (LSC) classification, described by McCormack in 1994 [2], is based on the shared constraints on the fractured vertebra. The objective of this classification is the prediction of the resistance of the anterior column, which, when considered insufficient, may require an earlier time. According to Parker JW *et al.* [13], this classification, whose total score is between 3 and 9 points, makes it possible to perform a short segment fixation taking one level above and one level below the fractured vertebra if the score is less than 7 points. However, Elzinga M *et al.* [14] questions this classification and its therapeutic interest. In fact, he brought in three observers for 40 thoracolumbar fractures to determine the load-sharing score (LSS) twice. He used Cohen's Κ (kappa) test, and found great variability between different observers, and even within the same observer. In agreement with Elzinga M *et al.* [14], the present work highlights the inadequacies of this classification which neglects the importance of load distribution on the vertebral body without taking into account the state of the complex posterior ligament or of the neurological status. Altay M *et al.* [15] thinks that this classification is not enough to choose between a short segment fixation and a long segment stabilization and proposes an association between the classification of Load Sharing and that of Magerl. In fact, Katonis P *et al.* [16] argues that a short segment fixation is sufficient in the lumbar region and constitutes a solid device without loss of correction; while Eno J-JT *et al.* [6] and Defino HLA *et al.* [17] used mono-segmental fixation with a graft in fractures with healthy pedicles. In our series, the average operating time was 99.3 minutes, which is close to that of Steib J-P *et al.* [18] (100 min) and Li X *et al.* [7] (101 min).

The comparison of bleeding abundance between the two types of segmental stabilization has been reported in several publications [19, 20], showing the advantage of short segment fixation. On the other hand, the length of hospital stay is 12.2 days on average for our series, comparable to that of Sanderson PL *et al.* [21] (12.4 days) and less significant than that of Sasso RC *et al.* [22] (17 days). According to the Denis Pain Scale, we only have 3 patients (10%) with a grade ≥3. Xu B *et al.* [23] and Shin T-S *et al.* [24] noted good functional results with only 5% having a grade ≥3. As for the evaluation of the functional capacity according to the Oswestry questionnaire, the average functional handicap score (ODI) is 14% for our series and is higher than 40% only in 3.3% of cases. This score is close to that reported by Steib J-P *et al.* [18] with an average ODI = 20.7%. In the literature, some consider that the posterior short segment fixation is insufficient to stabilize fractures of the thoracolumbar spine [25] and lacks solidity especially for certain types of fracture running the risk of a significant loss of correction and even dismantling of implant [26].

El-Shehaby A *et al.* [27] reported the failure of implant in 5 patients (10.9%): four treated by a short segment fixation and one treated by a long segment fixation. Also Xu B *et al.* [23] noted the presence of instrumentation failure in 16.2% of the cases occurring in the group treated by a short osteosynthesis. Some authors have attributed the implant failures to technical or instrumental failure. Other authors have considered that any breaking or bending of the implant was in favor of a pseudarthrosis [28]. Compared to other series in the literature using GSKD in the evaluation of loco-regional deformation of their fractures, our relative post-operative gains were close to those of Katonis P *et al.* [16] and less important than those found by Steib J-P *et al.* [18]. As for the study of the overall balance, the evaluation made according to the sagittal site in T9 and the VAS, carried out in 24 patients who could stand upright, made it possible to properly analyze the repercussions of the posterior arthrodesis on global spinal statics. Only 33.3% of the patients were balanced at the last retrospect. From this radiological analysis, we conclude that the short segment fixation badly corrects an imbalance which may be preexisting initially in these patients since computed tomography (CT) scans revealed certain cases of Shuermann's disease in our population.

## Conclusion

Short segment fixation makes it possible to minimize the extent of the skin incision and to limit not only the operating time, the abundance of the bleeding and the damage of soft tissues [29, 30], but also the duration of hospitalization [19, 20, 22] and therefore the costs of the stay and the care provided. In addition, stabilization with a short segment fixation means the use of only four screws instead of eight screws for the long segment fixation, which greatly reduces the cost of implant and therefore the cost of the intervention without incurring a significant loss of correction or a high rate of instrumentation failure.

### What is known about this topic

- Thoracolumbar fractures are common;
- Thoracolumbar fractures require osteosynthesis with a posterior and posterolateral graft;
- The choice of the type of stabilization remains a subject of controversy.

### What this study adds

- The short segment minimizes the extent of the skin incision;
- The short segment limits the damage of soft tissues;
- Prescribing the short segment fixation makes it possible for consolidation and arthrodesis of thoracolumbar fractures without incurring a significant loss of correction or a high rate of instrumentation failure.

## Competing interests

The authors declare no competing interests.
